# Real-time shear wave ultrasound elastography: a new tool for the evaluation of diaphragm and limb muscle stiffness in critically ill patients

**DOI:** 10.1186/s13054-020-2745-6

**Published:** 2020-02-03

**Authors:** Aurelien Flattres, Yassir Aarab, Stephanie Nougaret, Fanny Garnier, Romaric Larcher, Mathieu Amalric, Kada Klouche, Pascal Etienne, Gilles Subra, Samir Jaber, Nicolas Molinari, Stefan Matecki, Boris Jung

**Affiliations:** 1 Medical Intensive Care Unit, Montpellier University and Montpellier Lapeyronie Teaching Hospital, Avenue du Doyen Gaston Giraud, 34000 Montpellier, France; 2 INSERM U1046, CNRS UMR9214, Université de Montpellier, Montpellier, France; 3 IRCM, INSERM U1194, and Department of Radiology, Montpellier Cancer Research Institute, 208 Ave des Apothicaires, 34295 Montpellier, France; 4 Laboratoire Charles Coulomb (L2C), University of Montpellier, CNRS, Montpellier, France; 5 Institut des Biomolécules Max Mousseron (IBMM), UMR5247 CNRS, ENSCM, Université de Montpellier, 34000 Montpellier, France; 6 Saint Eloi Anesthesiology and Critical Care Medicine, Montpellier University and Montpellier Teaching Hospital, Montpellier, France; 7 Biostatistics Department, Montpellier University and Montpellier Teaching Hospital, Montpellier, France

**Keywords:** Sonoelastography, Intensive care unit acquired weakness, Diaphragmatic dysfunction, Cachexia

## Abstract

**Background:**

Muscle weakness following critical illness is the consequence of loss of muscle mass and alteration of muscle quality. It is associated with long-term disability. Ultrasonography is a reliable tool to quantify muscle mass, but studies that evaluate muscle quality at the critically ill bedside are lacking. Shear wave ultrasound elastography (SWE) provides spatial representation of soft tissue stiffness and measures of muscle quality. The reliability and reproducibility of SWE in critically ill patients has never been evaluated.

**Methods:**

Two operators tested in healthy controls and in critically ill patients the intra- and inter-operator reliability of the SWE using transversal and longitudinal views of the diaphragm and limb muscles. Reliability was calculated using the intra-class correlation coefficient and a bootstrap sampling method assessed their consistency.

**Results:**

We collected 560 images. Longitudinal views of the diaphragm (ICC 0.83 [0.50–0.94]), the biceps brachii (ICC 0.88 [0.67–0.96]) and the rectus femoris (ICC 0.76 [0.34–0.91]) were the most reliable views in a training set of healthy controls. Intra-class correlation coefficient for inter-operator reproducibility and intra-operator reliability was above 0.9 for all muscles in a validation set of healthy controls. In critically ill patients, inter-operator reproducibility and intra-operator 1 and 2 reliability ICCs were respectively 0.92 [0.71–0.98], 0.93 [0.82–0.98] and 0.92 [0.81–0.98] for the diaphragm; 0.96 [0.86–0.99], 0.98 [0.94–0.99] and 0.99 [0.96–1] for the biceps brachii and 0.91 [0.51–0.98], 0.97 [0.93–0.99] and 0.99 [0.97–1] for the rectus femoris. The probability to reach intra-class correlation coefficient greater than 0.8 in a 10,000 bootstrap sampling for inter-operator reproducibility was respectively 81%, 84% and 78% for the diaphragm, the biceps brachii and the rectus femoris respectively.

**Conclusions:**

SWE is a reliable technique to evaluate limb muscles and the diaphragm in both healthy controls and in critically ill patients.

**Trial registration:**

The study was registered (ClinicalTrial NCT03550222).

## Background

Survivors of critical illness have severe long-term functional disability [1]. Following the intensive care unit (ICU) stay, the quality of life and return to pre-ICU admission daily activities are impaired [1–3]. Both diaphragm and limb muscles are affected [1, 4–7]. Limb muscle weakness is one of the greatest issues linked with disability and poor outcomes in survivors [1, 8–11]. Diaphragm weakness has also been associated with poor outcome such as dependency on mechanical ventilation, prolonged ICU length of stay and mortality [4, 12–14]. Muscle weakness is related to muscle mass but beyond mass, loss of muscle function and muscle quality without atrophy has been observed in the elderly and in chronic hemodialysis patient populations, [15, 16] a condition labeled as dynapenia. At the ICU bedside, ultrasonography (US) is an inexpensive, user-friendly, non-volitional and non-invasive tool widely used by intensivists. US has been suggested to be a reliable tool to approximate muscle mass by measuring the cross-sectional area and muscle thickness [7, 17] and to also approximate muscle quality by grey-scale analysis of the muscle echogenicity [18, 19]. However, subcutaneous tissue and muscle edema and inflammation, frequently observed in the critically ill, can make the grey-scale analysis difficult to interpret. Moreover, diaphragm contractions during spontaneous breathing and passive stretch during mechanical ventilation may also make ultrasonography more difficult to perform in the critically ill patients than in healthy controls able to block their breathing cycle.

Shear wave ultrasound elastography (SWE) is a method of US imaging based on the detection of shear wave propagation through the tissue. By using inversion algorithms, this method maps the waves into elastograms and determines stiffness of the tissue by measuring the shear modulus value [20]. SWE has been established as an excellent diagnostic method for liver fibrosis, breast cancer and thyroid cancer [21]. In skeletal muscles, it provides a spatial representation and quantifiable measurement of the mechanical properties and an approximate of the muscle fibrosis and the activity of the disease in chronic myopathy [22] as well as a surrogate of diaphragm force production in healthy volunteers during isovolumetric inspiratory efforts [23]. Although SWE muscle analysis may provide new data about muscle quality during critical illness, it has never been evaluated in the very specific critically ill population which is exposed to severe structural muscular alterations. We therefore designed the present study with the aim of determining the reliability and reproducibility of SWE measurements for limb muscles and the diaphragm in both healthy subjects and in critically ill patients.

## Methods

### Study design and participants

We conducted the ULTRAMUSCLE prospective observational study at a medical ICU of the university hospital of Montpellier, France. Institutional ethical approval was obtained (2017-CLER-MTP-09-16), and the study was registered (ClinicalTrial NCT03550222). Because the ULTRAMUSCLE study was part of an image databank storage, consent was waived according to French law. We followed the STROBE guidelines for observational studies [24].

We enrolled 31 adult healthy subjects and 12 consecutive adult critically ill patients. Inclusion criteria for the critically ill patients were at least one organ failure (organ failure being defined by a Sepsis-related Organ Failure Assessment (SOFA) score [25] equal or greater than 3 for the organ considered) and an expected length of ICU stay of at least 3 days. Non-inclusion criteria were pregnancy, a history of neuromuscular disease (myasthenia gravis, chronic myopathy or neuropathy), spinal cord injury, recent intracranial disease, transfer from another ICU, age below 18 and refusal to participate in the trial. We first enrolled 16 healthy subjects as a training set to assess inter-operator reproducibility for each view and to determine the best view (longitudinal vs transversal) for the biceps, the rectus femoris and the diaphragm. We then enrolled an additional validation set of 15 healthy subjects to assess both inter- and intra-operator reliability for each muscle. Then we enrolled 12 consecutive critically ill patients during the last 2 weeks of November 2017, to assess both the inter- and intra-operator reliability for each muscle using the best views determined in healthy controls.

### Shear wave elastography imaging procedure

#### General procedure

An Aixplorer ultrasonic scanner (SuperSonic Imagine, Aix-en-Provence, France) was used with a 4–15 MHz linear transducer (SL15-4; SuperSonic Imagine) in SWE mode with musculoskeletal pre-set. All biceps, rectus femoris and diaphragm images were performed by two operators: an expert with 4 years of experience in the field of skeletal muscle ultrasound in the ICU and a novice who was not familiar with muscular ultrasound. Both were trained by the SuperSonic Imagine engineer before enrolling the first participant. All acquired images and data were stored and analysed secondarily as recommended by Nijholt et al. using Osirix DICOM Viewer software (Pixmeo, Geneva, Switzerland) [26].

#### Image acquisition

Healthy controls and patients were assessed in supine position, with their knees in passive extension and neutral rotation, and their arms by their sides and with forearms supinated and elbows at 30° of flexion. Conscious subjects were instructed to remain relaxed, to breathe as quietly as possible throughout the procedure, and to maintain an apnea at functional residual capacity for SWE acquisition. For mechanically ventilated patients, an end-expiratory pause was applied during SWE acquisition and the absence of diaphragm contraction was assessed using ventilator curves and ultrasound real-time images. The absence of movements and limb muscle contraction was also clinically carefully checked in the critically ill patients. We collected all measurements in triplicate, from the three muscles on the right side and at a resting position: diaphragm, biceps brachii and the rectus femoris. To enable repeat ultrasound assessments at the same location, a mark was drawn on the subject’s arm, leg and chest during the first measurement procedure, and was used by the two operators for all measurements. During image acquisition, transducers were placed with minimal compression on top of a generous amount of coupling gel to avoid distortion of the underlying tissue [27].

For all muscles, we collected both a transversal view that refers to the transducer being positioned perpendicular to the fibers and a longitudinal view that refers to the transducer being positioned parallel to the fibers. For the diaphragm views, the transducer was placed at the zone of apposition at the 8th–10th intercostal space between the right anterior and midaxillary lines to better identify the three-layered structure. All diaphragm views were acquired at the end of expiration when the muscle is the thinnest. All peripheral muscle images were acquired after 5 s of motionlessness assessed clinically. For the biceps brachii views, the transducer was placed perpendicular to the long axis of the arm on its anterior surface, over the mid-distance of the long head of the biceps brachii. For the rectus femoris images, the transducer was placed perpendicular to the long axis of the thigh on its anterior surface, at three fifths of the distance from the anterior superior iliac spine to the superior patellar border.

#### Image analysis

In biomechanics, stiffness is defined by the proportional relationship between the stress (the external force or compression) and strain (deformation) applied to it. Transmission of a longitudinal pulse leads to tissue displacement, which is detected by pulse echo ultrasound and allow the measurement of the shear wave velocity [*V* in m s^−1^]. The shear wave velocity *V* is proportional to the shear modulus (*μ* expressed in kPa) using the formula: *μ* = *ρ*.*v*^2^ (where *ρ* is the tissue density, equals to 1000 kg m^3^ in the human body). Hard tissues have a higher *μ* and *v* than soft ones. We performed the measurements at the middle portion of each muscle belly avoiding tendons, aponeurosis and fascial tissues to avoid measurement biases. When activating the SWE mode, a color-coded box representing the region of interest was super-imposed on the image (Fig. 2). SWE images were continuously acquired at a sampling rate of 2 Hz during apnea at the end of expiration for healthy subjects, or during an end-expiratory pause for mechanically ventilated patients. An elastography real-time 3-s cine loop was stored. We then offline manually drew the widest region of interest possible (labeled as a “Q-box Trace”) in a homogenous frozen image to average the shear modulus measurement into the drawn Q-box and to carefully exclude the surroundings tissue from the measurement.

#### Statistical analysis

Descriptive statistics are reported as numbers (%), mean ± standard deviation (SD) or median (and interquartile [25–75%]). Kolmogorov-Smirnov test was used to test normality. Differences in mean shear modulus (kPa) were compared using Student’s *t* test, Mann-Whitney test or Wilcoxon matched pairs signed rank test, as appropriate. Between-operator reproducibility and within-operator reliability were calculated using an analysis of variance intra-class correlation coefficient (ICC). An ICC is measured on a scale of 0 to 1; 1 represents perfect reliability with no measurement error, whereas 0 indicates no association. Measurement reliability was classified as follows: 0–0.49 “poor agreement”, 0.50–0.74 “moderate agreement”, 0.75–0.89 “good agreement”, 0.90–1 “excellent agreement”. The best ICC measured in the training set in the healthy controls was used to perform the measurements in both the validation set in the healthy controls and in the critically ill patients. To look for a minimum ICC value of 0.8 with an 80% power and the significance level set at *P* < 0.05, we calculated that 56 pairs of measures would be necessary for each muscle. Because this is the first study performed in the critically ill by intensivists and taking into the risk of uninterpretable images, we chose to increase this number by 25% (70 measures). The 31 healthy controls cohort was split into a training and a validation set (84–86 measures) and we enrolled 12 consecutive critically ill patients (68–72 measures). Finally, we used a bootstrap sampling method to assess the consistency of our findings. Statistical analysis was conducted using GraphPad, Prism (GraphPad Software, La Jolla, CA, USA) and R software version 3.5.1 (https://www.r-project.org/).

## Results

We collected 560 US examinations in healthy controls and in critically ill patients. Clinical characteristics are reported in Table 1. In the training set obtained from 16 healthy controls, we compared 192 SWE measurements collected from transversal and longitudinal images in the diaphragm, the biceps brachii and the rectus femoris by the two operators. Figure 2 is an illustration of the images obtained for the three evaluated muscles. The training set allowed us to determine that the best inter-operator reliability was obtained with the longitudinal views for the diaphragm and the biceps brachii. No significant difference in inter-operator reliability was observed between the transversal and the longitudinal views for the rectus femoris (Table 2).

**Table 1:** Baseline patient and healthy subject characteristics

Characteristics	Healthy subjects (*n* = 31)	Critically ill patients (*n* = 12)
Clinical characteristics		
Age (years)	26.7 ± 4.6	66.6 ± 20.9
Gender (male)	17 (54.8)	7 (58)
Body mass index (kg/m^2^)	22.6 [19.9–26.3]	25.2 [15.5–32.8]
SAPS II	–	59.1 ± 25.4
SOFA at admission	–	8.1 ± 6.2
Primary reason for admission	–	
Respiratory distress		7 (57)
Coma		2 (17)
Sepsis		2 (17)
Acute kidney injury		1 (8)
Clinical management during the first week in ICU	–	
Use of continuous neuromuscular blockade infusion		5 (42)
Use of systemic steroids		5 (42)
Duration of sedation (days)		4 [2–7]
Duration of invasive mechanical ventilation (days)		5.8 [3.7–7]
Duration of controlled mechanical ventilation (days)		1.7 [0.9–7]
Cumulative fluid balance at day 3 (ml)		6740 ± 2828

*Abbreviations*: *ICU* intensive care unit, *RASS* Richmond Agitation Sedation Scale, *SAPS II* Severe Acute Physiology Score II, *SOFA* Sepsis-related Organ Failure Assessment, *IQR* interquartile range, *SD* standard deviation

*All data are presented as either *n* (%) for categorical variables or median [IQR] and mean (SD) for continuous variables. Healthy subjects were younger than critically ill patients (*p* < 0.05)

**Table 2:** Mean and inter-operator reproducibility of shear modulus measurements in healthy subjects for various probe positions (training set, *n* = 16)

Measurements	Healthy subjects (*n* = 16)				
	*n* measures	Operator 1 Mean (SD)	Operator 2 Mean (SD)	*t*-test* (*p* value)	Inter-operator reproducibility (ICC)
Diaphragm shear modulus—longitudinal view (kPa)	32	19.4 (6.2)	20.1 (7)	0.19	0.83 [0.50 to 0.94]
Diaphragm shear modulus—transverse view (kPa)	32	25.4 (7.3)	22.4 (6.3)	0.73	0.3 [−0.86 to 0.75]
Biceps brachii shear modulus—transverse view (kPa)	32	11.1 (3.6)	16.1 (6)	< 0.05	0.39 [−0.29 to 0.76]
Biceps brachii shear modulus—longitudinal view (kPa)	32	28.9 (8.1)	27.8 (6.3)	0.82	0.88 [0.67 to 0.96]
Rectus femoris shear modulus—transverse view (kPa)	32	23 (6.8)	24.6 (8.3)	0.21	0.82 [0.50 to 0.94]
Rectus femoris shear modulus—longitudinal view (kPa)	32	15 (4.9)	15.4 (5.4)	0.57	0.76 [0.34 to 0.91]

*Abbreviations*: *ICC* intra-class coefficient correlation, *kPa* kilopascals, *SD* standard deviation. ICC are presented with 95% confidence interval

**Fig. 1:**
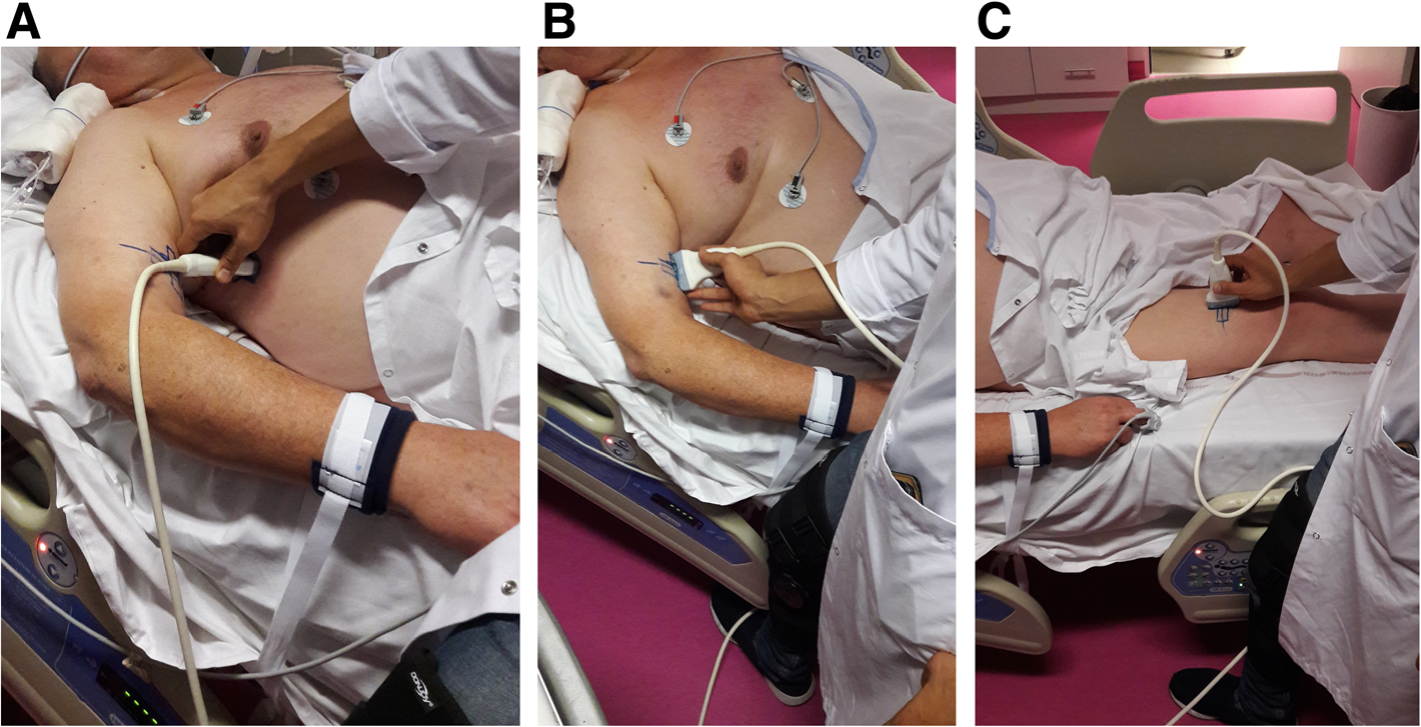
**a**–**c** Illustration showing the placement of the ultrasound probe

**Fig. 2:**
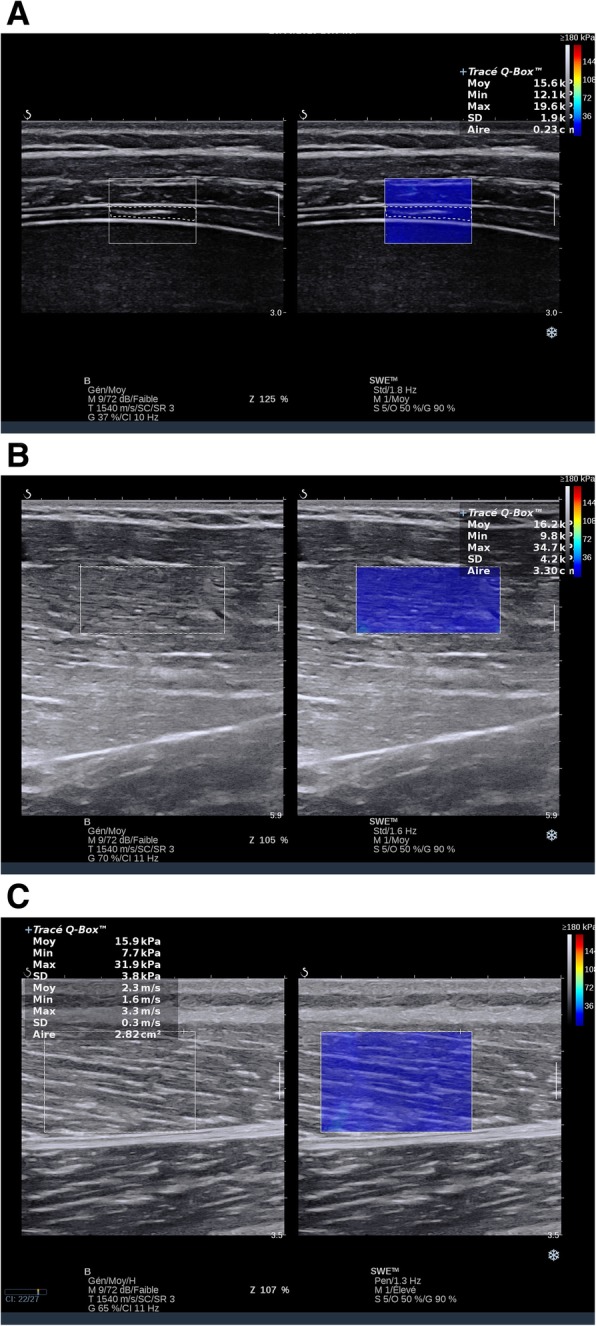
Representative elastograms for **a** diaphragm, **b** biceps brachii and **c** rectus femoris

**Fig. 3:**
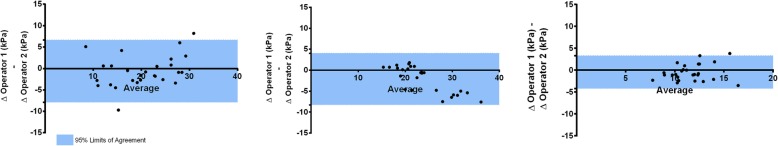
Bland-Altman plot of the difference of shear modulus between operators in healthy subjects

Figures 1 and 2 show how the images were obtained for the three evaluated muscles. In the validation set obtained from 15 healthy controls, 158 SWE measurements were analysed. Inter-operator and intra-operator reliability, measured on triplicates for each muscle were excellent, the median ICC being above 0.90 for each muscle for both the novice (intra-operator 2) and expert (intra-operator 1) operator (Table 3). Bland and Altman analysis confirmed the excellent inter and intra-operator reliability of the measurements (Fig. 3).

**Table 3:** Mean, inter-operator and intra-operator reliability of shear modulus measurements in healthy subjects (validation set, *n* = 15)

Measurements	Healthy subjects (*n* = 15)						
	Measures (*n*)	Operator 1 Mean (SD)	Operator 2 Mean (SD)	*t*-test (*p* value)	Inter-operator reproducibility (ICC)	Intra-operator 1 reliability (ICC)	Intra-operator 2 reliability (ICC)
Diaphragm shear modulus (kPa)	54	20 (7.3)	20.6 (6.1)	0.74	0.96 [0.85–0.99]	0.95 [0.82–0.99]	0.90 [0.70–0.98]
Biceps brachii shear modulus (kPa)	52	22.7 (4.3)	24.8 (6.8)	0.19	0.91 [0.54–0.98]	0.95 [0.83–0.99]	0.96 [0.87–0.99]
Rectus femoris shear modulus (kPa)	52	11.3 (2.4)	11.8 (2)	0.41	0.97 [0.77–0.99]	0.92 [0.70–0.98]	0.95 [0.82–0.99]

*Abbreviations*: *ICC* intra-class coefficient correlation, *kPa* kilopascals, *SD* standard deviation. ICC are presented with 95% confidence interval

**Fig. 4:**
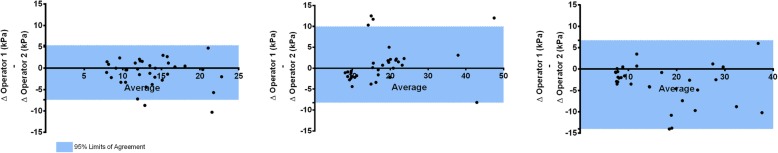
Bland-Altman plot of the difference of shear modulus between operators in critical ill patients

We then enrolled 12 critically ill patients (Table 1) and analysed 210 images. Global shear modulus in kPa was 13.7 (± 4.4), 17.8 (± 9.4) and 16.6 (± 9.6) for diaphragm, biceps brachii and rectus femoris respectively. No significant differences were found between the novice and expert operators as shown in the Bland-Altman graphs (Fig. 4). The average inter-operator reproducibility was excellent for the three muscles with ICCs being above 0.90. The intra-operator reliability of the SWE measurements was also excellent, ICC being above 0.9 (Table 4). The inter-operator reliability of the SWE measures was assessed using bootstrap resampling with replacement (10,000 samples) to the measures performed on the 12 critically ill patients. The probability to reach good to excellent ICCs between operators (above 0.8) for diaphragm, biceps brachii and rectus femoris in a 10,000 bootstrap sample was 81%, 84% and 78% respectively.

**Table 4:** Mean, inter-operator and intra-operator reliability of shear modulus measurements in critical ill patients (*n* = 12)

Measurements	Critically ill patients (*n* = 12)						
	Measures (*n*)	Operator 1Mean (SD)	Operator 2 Mean (SD)	*t*-test (*p* value)	Inter-operator reproducibility (ICC)	Intra-operator 1 reliability (ICC)	Intra-operator 2 reliability (ICC)
Diaphragm shear modulus (kPa)	72	13.1 (4.2)	14.2 (4.6)	0.31	0.92 [0.71–0.98]	0.93 [0.82–0.98]	0.92 [0.81–0.98]
Biceps shear modulus (kPa)	70	18.2 (10)	17.4 (8.9)	0.71	0.96 [0.86–0.99]	0.98 [0.94–0.99]	0.99 [0.96–1]
Rectus femoris shear modulus (kPa)	68	14.7 (9)	18.4 (10.2)	0.11	0.91 [0.51–0.98]	0.97 [0.93–0.99]	0.99 [0.97–1]

*Abbreviations*: *ICC* intra-class coefficient correlation, *kPa* kilopascals, *SD* standard deviation. ICC are presented with 95% confidence interval

## Discussion

This study shows that intra- and inter-operator reliability of shear modulus evaluation, a parameter of muscle quality in limb muscles and the diaphragm in both healthy controls and in critically ill patients, is excellent. US evaluation of critically ill patients at the bedside has been largely adopted by intensivists because it is easily available, non-expensive and non-invasive and it can be used as a monitoring tool during the ICU stay. Therefore, besides its large indications in cardiovascular or respiratory management, it has also often been described to evaluate limb muscle and diaphragm mass in weak patients during the ICU stay [7, 28–32]. Very few US studies have however focused on parameters that reflect muscle quality. In a landmark study, Puthucheary et al. used grey-scaling of the rectus femoris images in 30 critically ill patients and reported that rectus femoris echogenicity variation could predict myonecrosis with an area under the receiver operating characteristic curve of 0.74 (95%CI, 0.565 to 0.919). Parry et al. reported in 22 critically ill patients that changes in vastus intermedius but not rectus femoris echogenicity over time were correlated with physical function tests collected at ICU awakening and at ICU discharge [28]. Beyond quantitative analysis of the muscle mass, non-invasive tools are urgently needed to provide measurable parameters of limb and respiratory muscle quality. Real-time non-volitional tools, available at the bedside such as SWE, would provide targets for clinical trials aiming at improving quality of life and autonomy in survivors of critical illness [33]. Because different pathologic and healthy tissues reveal a similar echogenicity pattern and similar grey-scale but different shear modulus values, sonoelastographic techniques such as SWE measurement might be a promising tool to evaluate and to monitor muscle quality and in vivo contractile property changes over time [27, 34]. For instance, high shear modulus is associated with muscle stiffness in cerebral palsy or in late Duchenne’s myopathy [35], while low shear modulus is associated with atrophy in a GNE chronic myopathy [22]. Moreover, shear modulus but not echogenicity is associated with muscle fibrosis. No study has ever been performed to evaluate shear modulus measurement feasibility and reliability in the critically ill population at high risk of muscle edema.

We report herein that shear modulus measurement was reliable and easy to learn for both expert and novice non-radiologist operators working with critically ill patients. Few others have assessed inter-operator and intra-operator reliability in measuring muscle shear modulus and have reported similar results. Tas et al. reported in 12 healthy subjects an excellent intra-operator reliability and inter-operator reproducibility for rectus femoris (ICC above 0.9) [36]. Dorado-Cortez et al. reported better reliability and reproducibility for shear wave velocity measured in longitudinal plane than transverse plane in healthy lower limb muscles [37]. Du et al. found a good agreement for shear modulus reproducibility in patients with Parkinson’s disease, and a significant difference in biceps brachii stiffness between healthy controls (24 ± 5 kPa), mildly symptomatic patients (48 ± 24 kPa) and remarkably symptomatic patients (60 ± 21 kPa) [38].

To assess the muscle globally and to avoid intramuscular heterogenous measurements, we chose to use the largest square possible as the region of interest.

In the present study, we explored both the limb muscles and the diaphragm, the main inspiratory muscle. Nowadays, since specific phrenic nerve stimulation tools to explore critical illness-associated diaphragm weakness are not routinely available at the bedside [4, 12] (and diaphragmatic biopsies are very difficult to obtain in humans [5, 39, 40]), non-invasive tools to explore the diaphragm using US is more and more popular. US may help by approximating diaphragm mass by measuring thickness as well as approximating diaphragm contractile activity by measuring the excursion and the thickening fraction [41]. A pioneer study by Goligher has reported that during critical illness and mechanical ventilation, the diaphragm thickness may decrease (supposedly because of atrophy), but may not change during the stay or may increase (supposedly because of myotrauma) [30, 42]. Since diaphragmatic shear modulus has been associated with diaphragmatic function in spontaneously breathing volunteers [43], shear modulus analysis of the diaphragm in the critically ill may pave the way towards a non-invasive better understanding of diaphragm quality changes during critical illness. Our study presents limits that should be discussed. First, we did not perform muscle biopsies to correlate shear modulus to pathology. However, most of the studies that have evaluated SWE in muscles did not show pathology data either [27, 34]. Second, precautions must be taken into consideration when shear modulus is measured. Position of the patient, muscle contraction and pressure applied to the muscle by the probe do indeed influence the shear modulus value [27, 34, 44]. We therefore took extreme precautions to limit any measurement bias and used an excessive amount of gel, did not apply any pressure to the muscle during the evaluation and used triplicates to calculate the mean shear modulus. We also paid extra attention to measure the shear modulus in muscles with no contractions and at the end of the expiration for the diaphragm. In the healthy controls, we report a diaphragm shear modulus above the range observed by Bachasson et al. [23]. Two main differences should be noted between the two studies. We performed the measurements in the supine position rather than the semi-recumbent position which may have impaired the diaphragm to completely relax at the end of the expiration. We designed the present study as a real-life bedside study and did not use a pneumotachograph or a transdiaphragmatic pressure monitoring. Therefore, even after taking extra precautions to measure the shear modulus at the end of expiration with no diaphragm contraction, it is possible that some of the measurements were performed above the functional residual capacity. However, because both intra-operator reliability and inter-operator reproducibility ICC were very high, our results suggest that shear wave elastography is a feasible technique to describe diaphragm ultrastructure. Diaphragm biopsies using critically ill animal models may help assessing the accuracy of the shear wave elastography technique in phenotyping diaphragm weakness. To limit the influence of muscle anisotropy on the measured value, we chose to use a large square as the region of interest to measure the signal. Third, we used shear modulus measurement rather than shear wave velocity. All commercially available US elastography systems are based on the prerequisite assumption that the material is elastic, incompressible, homogenous and isotropic, with a density which equals 1 g/cm^3^. The muscle tissue is however anisotropic and shear modulus should therefore be used as a variation during the course of the disease.

## Conclusion

Our study shows for the first time that a non-volitional, non-invasive ultrasonographic evaluation of muscle stiffness and contractile properties using shear modulus measurement is reliable in the critically ill population for both the limb muscles and the diaphragm. SWE is an US tool that should be investigated in a larger population of critically ill patients to assess whether it might serve as a tool to identify different patient’s phenotypes of muscle weakness.

## Data Availability

The authors of this manuscript consent to share the collected data to others. Data are provided to qualified investigators free of charge. Required documents to request data include a summary of the research plan, request form and IRB review. Datasets will be shared after careful examination by the study board of investigators. Data will be available 12 months after the main publication and indefinitely.

## Abbreviations

ICC: Intra-class correlation coefficient
ICU: Intensive care unit
SD: Standard deviation
SOFA: Sepsis-related Organ Failure Assessment
SWE: Shear wave ultrasound elastography
US: Ultrasonography
μ: Shear modulus
