# Correction to: Real-time shear wave ultrasound elastography: a new tool for the evaluation of diaphragm and limb muscle stiffness in critically ill patients

**DOI:** 10.1186/s13054-020-2802-1

**Published:** 2020-03-05

**Authors:** Aurelien Flatres, Yassir Aarab, Stephanie Nougaret, Fanny Garnier, Romaric Larcher, Mathieu Amalric, Kada Klouche, Pascal Etienne, Gilles Subra, Samir Jaber, Nicolas Molinari, Stefan Matecki, Boris Jung

**Affiliations:** 1 Medical Intensive Care Unit, Montpellier University and Montpellier Lapeyronie Teaching Hospital, Avenue du Doyen Gaston Giraud, 34000 Montpellier, France; 2 INSERM U1046, CNRS UMR9214, Université de Montpellier, Montpellier, France; 3 IRCM, INSERM U1194, and Department of Radiology, Montpellier Cancer Research Institute, 208 Ave des Apothicaires, 34295 Montpellier, France; 4 Laboratoire Charles Coulomb (L2C), University of Montpellier, CNRS, Montpellier, France; 5 Institut des Biomolécules Max Mousseron (IBMM), UMR5247 CNRS, ENSCM, Université de Montpellier, 34000 Montpellier, France; 6 Saint Eloi Anesthesiology and Critical Care Medicine, Montpellier University and Montpellier Teaching Hospital, Montpellier, France; 7 Biostatistics Department, Montpellier University and Montpellier Teaching Hospital, Montpellier, France

**Correction to: Crit Care (2020) 24:34**

**https://doi.org/10.1186/s13054-020-2745-6**

In the publication of this article [1], there was an error in the Family Name of one of the authors. This has now been updated in the original article.

The error: Flattres

Should instead read: Flatres

## References

[1] Flatres A, Aarab Y, Nougaret S (2020). Real-time shear wave ultrasound elastography: a new tool for the evaluation of diaphragm and limb muscle stiffness in critically ill patients. Crit Care.

